# Translating Pharmacogenetics and Pharmacogenomics to the Clinic: Progress in Human and Veterinary Medicine

**DOI:** 10.3389/fvets.2019.00022

**Published:** 2019-02-11

**Authors:** Deirdre P. Campion, Fiona J. Dowell

**Affiliations:** ^1^UCD School of Veterinary Medicine, University College Dublin, Dublin, Ireland; ^2^Division of Veterinary Science and Education, School of Veterinary Medicine, College of Medical, Veterinary and Life Sciences, University of Glasgow, Glasgow, United Kingdom

**Keywords:** veterinary medicine, pharmacogenetics, pgx, pharmacogene, gene biomarkers, personalized medicine

## Abstract

As targeted personalized therapy becomes more widely used in human medicine, clients will expect the veterinary clinician to be able to implement an evidence-based strategy regarding both the prescribing of medicines and also recognition of the potential for adverse drug reactions (ADR) for their pet, at breed and individual level. This review aims to provide an overview of current developments and challenges in pharmacogenetics in medicine for a veterinary audience and to map these to developments in veterinary pharmacogenetics. Pharmacogenetics has been in development over the past 100 years but has been revolutionized following the publication of the human, and then veterinary species genomes. Genetic biomarkers called pharmacogenes have been identified as specific genetic loci on chromosomes which are associated with either positive or adverse drug responses. Pharmacogene variation may be classified according to the associated drug response, such as a change in (1) the pharmacokinetics; (2) the pharmacodynamics; (3) genes in the downstream pathway of the drug or (4) the effect of “off-target” genes resulting in a response that is unrelated to the intended target. There are many barriers to translation of pharmacogenetic information to the clinic, however, in human medicine, international initiatives are promising real change in the delivery of personalized medicine by 2025. We argue that for effective translation into the veterinary clinic, clinicians, international experts, and stakeholders must collaborate to ensure quality assurance and genetic test validation so that animals may also benefit from this genomics revolution.

## Introduction

Pharmacogenetics has been defined in human medicine as the “variability due to hereditary” (1) and as such, the concept pre-dates the discoveries of the Human Genome project. The term “pharmacogenomics” initially was used to describe the development of new drugs based on new understanding provided by genomic research, and although the terms “pharmacogenetics” and “pharmacogenomics” are often used interchangeably (1), “pharmacogenomics” is considered to focus on the “expressed genome” and techniques to study drug responses include proteomics, metabolomics, and gene expression (2). To encompass both concepts, the abbreviation PGx has come into use, defined as “the individualization of drug therapy through medication selection or dose adjustment based upon direct (e.g., genotyping) or indirect (e.g., phenotyping) assessment of an individual's genetic constitution for drug response” Swen et al. (3). A closely linked concept is “personalized medicine” which has been redefined for the modern era as “the use of combined knowledge (genetic or otherwise) about a person to predict disease susceptibility, disease prognosis, or treatment response and thereby improve that person's health (4).”

Since drugs have first been used in man, it has been recognized that certain individuals respond differently to certain drugs than others do. These differing responses may now be attributed to the individual's genotype. With increased understanding of genetic differences in human populations, and between individuals, the concept of “precision medicine” is gaining currency. In Europe, the recently established European Commission-funded Ubiquitous-Pharmacogenomics Consortium (U-PGx), aims to make personalized medicine prescribing accessible to every European citizen (5).

Within the veterinary sphere, except for very specific broadly understood genetic mutations such as MDR-1/ABCD-1 mutation in canine herding breeds, the direct application of PGx to clinical prescribing has been limited to date. However, as targeted medicine becomes more widely used in human medicine, clients may expect that the veterinary clinician should also be able to implement an evidence-based strategy regarding both the prescribing of medicines and also recognition of the potential for adverse drug reactions (ADR) for their animal, at breed and individual level.

This review aims to provide an overview of the current developments and challenges in PGx in medicine for a veterinary audience, to map to and expand on these with respect to current PGx knowledge and potential developments in veterinary pharmacotherapeutics.

## Genetic Variation in Man and Domestic Species

Responses to drugs and adverse reaction to drug treatment, in both humans and animals are influenced by a number of factors such as age, gender, environment, and by an individual's genetic make-up which may be influenced by ethnicity or in the case of animals, breed. Genetic variants arise within a species as a result of DNA base pair substitutions resulting in single nucleotide polymorphisms (SNPs) or as a result of either an insertion or deletion in a sequence (indels). SNPs are relatively common and are considered to occur in approximately 1 in 1,000 base pairs in the human genome (6). SNPs and indels, if located in the coding region of genes, may have significant effects on key protein production within cells (6).

A change in genetic variant frequency at population level, or “genetic drift,” occurs naturally as a result of “bottleneck” events, when populations fall below a certain level and subgroup of a species are cut off from each other, with the net result that certain genetic variants alleles could become “lost”; conversely, certain variants could become predominant. Variation may also occur as a result of the selection pressures which different populations are subjected to, for example, in humans, the heritable trait glucose-6-phosphate dehydrogenase (G6PD) deficiency confers a relative degree of protection against the malaria parasite and therefore variations in G6PD are found to a greater extent in populations whose ancestry derives from tropical regions such as Sub-Saharan Africa and Southern Asia (7). Finally, a genetic variation may arise naturally within a population, and a variant allele may subsequently persist in that population as its effect is generally neutral on normal survival and reproduction (8).

Domestication and man's subsequent use of animals for specific purposes has led to selective breeding based on phenotypic characteristics such as production traits in farmed species, size, speed, and/or endurance in beasts of burden, and behavior and aesthetics in companion animals, with the resulting creation of subpopulations within the different domestic species such that modern breeds may bear little resemblance with the ancient ancestor. This divergence is possibly the most extreme in the domestic dog, *Canis familiaris* (9). Phylogenetic analysis of the whole canine genome indicates that the modern domestic dog is descended from the Eurasian gray wolf (*Canis lupus lupus*), with the primary divergence occurring more than fifteen thousand years ago. This genetic drift is considered to be as a result of two “bottleneck” events, believed to have occurred during domestication (10). More recently, breed-specific bottlenecks have occurred, where canine breeds were created from a small population of “founder” individuals with the preferred phenotype, followed by inbreeding within that limited population (10). This selection for different extreme phenotypes within canine breeds by far exceeds artificial selection pressures within other domestic animals, and currently, the UK Kennel Club lists 221 canine breeds (11). This selection pressure has the potential to lead to a higher proportion of genetic variants within specific dog breeds, which might not occur in semi-feral or “village” dog populations (12).

As a comparison, the domestic cat remains largely phenotypically similar to its ancestor, *Felis silvestris*, as this species has not undergone the extreme selection pressures of other domesticated species (13). The Cat Fanciers' Association currently lists 42 cat breeds (14), of which 16 are “natural breeds” and are thought to be from historical populations arising in specific geographical locations, while the remaining breeds were developed through relatively recent deliberate selection, or “discovered” in feral or domestic populations over the past 50 years, and are usually defined as simple, single-gene variants, such as genes for coat color and/or texture (13).

## Evolution of PGx in Human and Veterinary Medicine

Pythagoras is commonly credited with having first reported that the adverse effect of fava beans (*Vica fava*), now known to be acute haemolytic anemia, was associated with only some individuals. In the modern scientific era, Sir Archibald Edward Garrod is considered to be the first scientist to recognize inherited aspects of the metabolism of substances, with his description of alkaptonuria, a rare inherited condition resulting in black-colored urine due to an inability to metabolize ingested tyrosine and phenylalanine, discussed in his 1923 book “Inborn Errors of Metabolism” (15).

In the same decade, the 1920s, the anti-malarial drug 8-aminoquinoline (pamaquine) was reported to induce acute haemolytic anemia, primarily in African-Americans. The drug primaquine was found to have the same adverse effect, and again it was noted that African-Americans had a greater susceptibility in comparison to persons of European descent (16). This ADR and also the effect of fava beans were subsequently found to be due to glucose-6-phosphate dehydrogenase (G6PD) deficiency, which altered erythrocyte metabolism (17). In 1957, in a seminal edition of the Journal of the American Medical Association, Motulsky theorized that these adverse reactions were as a result of genetic traits (18). Shortly afterwards in 1957 at an international conference on modern problems in human genetics, Vogel for the first time coined the term “Pharmakogenetik” (19) establishing PGx as a distinct discipline.

Motulsky and collaborators went on to characterize the human genetic variation in the pseudocholinesterase enzyme associated with prolonged response to succinylcholine during anesthetic induction (20).

Inter-individual variabilities in the rate of drug metabolism were also being identified during the same timeframe as the above ADRs. In the mid to late 50's, isoniazid, an important drug for treatment of tuberculosis, was found to have a variation in the rate of metabolism in different ethnic groups- subjects were found to be either “rapid” or “slow inactivators”. It was found, for example, that the Japanese population contained a greater proportion of “rapid inactivators” than the Caucasian population. This was explained as a genetic variation in an acetylator enzyme (21), and 40 years later was specifically identified as a genetic variation in the gene encoding the metabolizing enzyme N-acetyltransferase-2 (NAT-2) (22).

From the 1960s through to the 1980s, a range of studies investigated inter-population and inter-individual variations in drug metabolism. Studies on the wide variability in metabolism of the antihypotensive agent debrisoquine led eventually to the identification, cloning, and characterization of the first known polymorphic drug-metabolizing enzyme -cytochrome P450 enzyme (CYP)2D6 (23). By the end of the 1980s, more than 100 examples of pharmacogenetic responses had been identified in the human, including exaggerated drug responses, lack of expected drug efficacy, or novel drug effects (24).

As these human investigations proceeded, the variability in metabolism was also being investigated in experimental animals, primarily laboratory rodents. For example, dark agouti (DA) rats were found to be “slow metabolisers” of debrisoquine in comparison to either Fischer or Lewis strains (25) and therefore the DA stain was used subsequently as a model of the CYP2D6 poor metaboliser phenotype (26).

Domestic animal species also served as experimental models for heritable human disease. The animal model for the heritable condition malignant hyperthermia (MH), a hypermetabolic adverse reaction to succinylcholine and volatile anesthetics (27), was first identified in 3 Landrace x cross pig littermates (28) although the breed was not identified in the initial paper. Subsequently, the Pietrain breed of pigs was found to have a high likelihood of MH susceptibility. It was noted that pigs susceptible to MH also have an increased likelihood of developing porcine stress syndrome (PSS), which affects meat quality and appearance (29) pointing to an issue relating to an excitable cell protein. The genes responsible for MH in pigs was subsequently identified as due to a ryanodine receptor 1 (RYR-1) mutation and RYR-1 gene polymorphisms have since been identified in a range of pig breeds (30). Following immediately on from this work in pigs, variation in the homolog gene, human RYR-1, was identified as a genetic biomarker for human MH (31).

Breed or population associated differences in drug responses in companion animals did not receive major attention until the emergence of the macrocyclic lactone ivermectin onto the market in 1981, originally only licensed for use in farmed species for external and internal parasites. Its effectiveness led to off-label use of the large animal formulation in dogs. By 1983, “ivermectin toxicity” was being reported in dogs, particularly Collie and herding breeds (32) so that by the time an ivermectin product was authorized for use in dogs in 1987 as a heartworm preventative, specific testing had been carried out to ensure that the dosage chosen was safe in what were termed “sensitive” breeds, specifically Collies (33). The role of P-glycoprotein as a blood-brain barrier “gatekeeper” was discovered as an “incidental finding” in experimental laboratory animals. When ATP binding cassette subfamily B member 1a (ABCB-1a, then named MDR-1a) knockout mice were treated with ivermectin for mite infestation, nearly all of the group died from neurotoxicity (34). Ivermectin toxicity in dogs was then subsequently identified as a deletion mutation of the ABCB-1 gene encoding P-glycoprotein (35).

The scientific endeavor which revolutionized all aspects of genetics and accelerated the rate of PGx discovery has been the Human Genome Project, with the first full genome sequenced in 2001 (36). The dog and cat draft genomes were published in 2005 (37) and 2007 (38) respectively. Domestic cattle (*Bos taurus*) (39) and horse (40) genome sequences were published in 2009.

The publication of these sequences has been accompanied by an improved array of technologies to interrogate the generated data and has contributed to the development of the discipline of PGx. Whereas historically the study of genetic responses both in human and veterinary medicine followed a “phenotype-to-genotype” approach, these tools shift the dynamic of exploration such that identified genomic variability may be scrutinized to identify phenotypic variability (41).

## PGx and Pharmacogenes

The tools available to the modern clinical scientist allow genetic loci of interest to be targeted for genomic investigation. These loci are often called “genomic biomarkers” and may be used to predict the likelihood that a person or animal may develop a disease condition, and therefore predict medical “risk” (42). By the same token, specific loci have been identified to be associated with drug responses, and have been named “pharmacogenes,” although it is now known that many drug effects are under the influence of multiple genes within the same patient.

A significant number of well-recognized “clinically actionable” pharmacogenes have been identified in human medicine, primarily encoding genes relating to pharmacokinetics (43). The term “clinically actionable” in this context is described as using knowledge of genetic variants, where there is clear evidence of an effect, for guiding clinical decisions to improve health outcomes (44).

PGx variances may be classified into groupings based on the drug response “differences” which impact on drug metabolism, efficacy, and risk of adverse effects. These group profiles are (1) “pharmacokinetic,” reflecting genes that influence absorption, distribution, metabolism, and elimination (ADME); (2) “pharmacodynamic”- genes that encode the intended target of the drug; (3) “pathway”- genes that encode proteins in the downstream pathway (i.e., metabolic or cellular) of the drug; and (4) “off-target”- genes that encode proteins that are not in the intended pathway (2); see Table 1.

**Table 1:** PGx variants determining drug responses.

**Class**	**Description**	**Example genes or gene products**	**Primary effect**	**Example gene biomarker and drug pair- human**	**Example gene biomarker and drug pair- veterinary spp**.	**Potential clinical approach**
Pharmacokinetic	Genes relating to ADME	Cytochrome P 450 (CYP) enzymes, drug transporters	Alter concentration of the active drug at the intended or at an unintended site of drug action	*CYP2C9* and warfarin (45) *CYP2D6* and codeine (46) *CYP2B6* and propofol (47)	Equine *CYP2D50* and tramadol (48) Canine *ABCB-1* and ivermectin (35)	Change drug dose; choose alternate drug with different ADME
Pharmacodynamic	Genes that encode the intended drug target	Cell surface receptors and intracellular enzymes	Alter the levels of the drug target or alter the ability of a drug to bind to drug target	*VKORC1* and warfarin (45)	Canine *OXTR* and oxytocin (49) Canine *PDE5A* and sildendafil (50)	Change drug dose; choose alternate drug with different target
Pathway	Genes that encode proteins in the downstream pathway of drug	Signaling molecules, cellular pathways, transcription factors	Alter the activity of the pathway targeted by drug therapy	*LDLR* and statins (51)	–	Change drug dose; choose alternate drug with different drug pathway
Off-target	Genes that encode proteins that are not in the downstream pathway of drug	Immune recognition proteins	Generate an immune or other response to drug or metabolite	*HLA-B* and abacavir (52)	MHC genes and idiosyncratic responses? Porcine (30), Canine (53) & Equine (54) *RYR1* and halothane/sevoflurane	Change drug dose; choose alternate drug with different chemical structure

*Adapted from Voora (2)*.

### Genes Involved in Pharmacokinetic Variation

Drug metabolism is characterized by two phases: (1) Phase I metabolism primarily carried out by Cytochome P 450 (CYP) hepatic microsomal enzymes and (2) Phase II metabolism which normally involves conjugation with an ionized group (55). A vast array of enzymes and pathways are involved in both phases, and significant species differences exist. Considering the complexity of the process of drug metabolism, it is not surprising that the genes involved in drug metabolism are also complex. Genes involved in pharmacokinetics are known to have multiple polymorphic variations both in man (56) and in veterinary species (48, 57). For example, the variability in response to warfarin, a drug used extensively in human medicine as an anticlotting agent, is influenced by both the gene encoding the cytochrome P450 complex subunit 2C9 (CYP2C9) for which there are 60 known human haplotypes, and also cytochrome P450 complex subunit 4F2 (CYP4F2) a primary liver vitamin K oxidase for which there are three haplotypes, and rs12777823, a SNP in the CYP2C cluster which is associated with reduced clearance in persons with African ancestry (45, 58). The case of warfarin is further complicated by pharmacodynamic genetic variation, specifically in the gene encoding the molecular target for warfarin, vitamin K epoxide reductase subunit 1 (VKORC1) (58) for which there are 13 known human haplotypes.

Although not as advanced as in human medicine, great strides have been made in the study of pharmacokinetic variation in veterinary species (59). Much of the current scientific understanding of CYP metabolism has been elucidated in humans and rodents, although in recent decades, exploration has commenced in veterinary species (57). It is important to note that interspecies differences in drug metabolism mean that information derived from human gene-biomarker: probe substance pairs do not automatically extrapolate to veterinary species. The genetics of canine CYP, extensively reviewed in 2013 (60) shows specific similarities but also significant differences from human CYP. There are species differences in gene sequences, and therefore even where there are clearly identifiable orthologs (genes derived from common and sometimes ancient ancestral genes) the names may differ. As an example, CYP2D15 is the canine ortholog of human CYP2D6 (61). Human CYP2D6, whose polymorphisms were first identified with debrisoquine, is considered be involved in the metabolism of over 25% of currently marketed drugs in humans, including antiarrhythmics, adrenoceptor antagonists, and tricyclic antidepressants (62). Canine CYP2D15 shares many, although not all therapeutic drug substrates with human CYP2D6 (57) but has a greater relative degree of expression in the normal canine the intestine and liver than CYP2D6 in normal human intestine and liver, which explains why drugs which are CYP2D16 substrates have lower oral bioavailability in dogs than in humans (60). Notwithstanding the importance of canine CYP2D15, to date no polymorphisms have been identified which have been definitively proven to affect canine drug metabolism (59).

This is not necessarily true of the horse, however, as there is initial evidence that the equine ortholog for human CYP2D6, the equine CYP2D50 gene, is polymorphic, and effects on drug metabolism have been identified (48). This study analyzed blood samples from only 150 horses, primarily Thoroughbreds, and identified 126 exonic SNPs, of which 31 appeared in more than one horse. A subset of the genotyped horses (23 Thoroughbreds, 1 Standardbred) were administered the known human CYP2D6 substrate tramadol by nasogastric tube, and subsequent plasma levels of tramadol were measured, using jugular samples taken over the following 96 hours. Although the number of animals used was very small, the resulting pharmacokinetic data is suggestive of specific CYP2D50 SNPs being associated with slower drug metabolism in horses, although further work is needed to confirm this.

One example where canine CYP polymorphisms have been identified is Canine CYP2B11, found primarily in Labrador retrievers, Collies, Uraguayan Cimarrons, Silken Windhound, Scottish Deerhounds, Greyhounds, and Welsh Corgis (59). Canine CYP2B11 is the canine ortholog of the human clinically actionable highly polymorphic pharmacogene CYP2B6, and is considered to act on a number of important substrates including propofol in both species (60). Although some of these breed-associated polymorphisms have not yet been associated with drug metabolism differences, the variant CYP2B11-H3, a single SNP in 3′ untranslated region of mRNA (59), may contribute to the previously identified reduction in metabolism of propofol in greyhound in comparison with mixed-breed canine hepatic microsomal preparations (63). Other known substrates of CYP2B11 include a range of drugs used frequently in veterinary practice, including atipamezole, diclofenac, ketamine, medetomidine, midazolam, and temazepam (60), and further evaluation of Canine CYP2B11 polymorphisms combined with hepatic microsomal studies may inform the therapeutic use of these drugs in the veterinary clinic.

Pharmacokinetic difference may also involve drug transporters, such as efflux transporters, which influence the distribution, effects, and elimination of drugs. The family of genes which encode for efflux transporters of the ATP-binding cassette (ABC) are of ancient phylogenic origin, are found in prokaryotes and eukaryotes, and where present and functioning, are responsible for mediating a range of therapeutic outcomes- from antimicrobial resistance in bacteria to failure of chemotherapy in humans (64). Efflux transporters serve a vital function to translocate substrates across cell membranes and lower intracellular concentrations. In mammals, efflux transporters are highly expressed in many organs such as the intestine, brain, liver, kidney, adrenals, placenta, and lungs, where their presence typically affects drug bioavailability, distribution and elimination (65).

In human medicine, ATP-binding cassette B-1 (ABCB-1), formerly known as the multi-drug resistance gene-1 (MDR-1), is a listed as a “very important” pharmacogene, a concept explained in section **Translating PGx to the clinic**, and is one of forty-nine putative gene members in the superfamily of ABC transporters that encode human cellular efflux pump P-glycoprotein (65). P-glycoprotein is expressed across a broad range of tissues and was first identified as a result of increased expression of this cell surface protein in cancer cell lines which were resistant to certain chemotherapeutics (66).

Substrates for P-glycoprotein in man and animals include a functionally diverse range of therapeutic compounds (65), and include anticancer agents such as doxorubicin and vincristine; steroid hormones such as hydrocortisone and dexamethasone; anti-histamines such as fexofenadine and cimetidine; opiates and derivatives such as morphine and loperamide; and cardiac drugs such as digoxin and quinidine (67, 68). Although functionally variant alleles have been identified in different populations of humans (69), as yet the drug substrate: gene pairs are not listed as “clinically actionable” by the US Food and Drug Administration (70).

The gene encoding P-glycoprotein in dogs and cats is also named ABCB-1, and in these species clinically relevant ABCB-1 polymorphisms have been identified. In dogs, as mentioned previously, the veterinary importance of ABCB-1 came to light when the anti-parasitic drug and P-glycoprotein substrate ivermectin was introduced to the market (35). One ABCB-1 mutation in dogs consists of a 4 base-pair deletion at the 5′ end, with the effect that p-glycoprotein synthesis is incomplete (35). Loss of p-glycoprotein function is most commonly associated with neurotoxicity and fatal toxicosis, as drugs which are normally prevented from entry into the brain by the blood-brain barrier may now enter and have a direct effect on brain neurons, as occurred with ivermectin to disastrous effect. One case report describes a Collie with ABCB-1 mutation which displayed severe neurologic signs, including disorientation, ataxia, rear limb weakness, when treated with oral loperamide at therapeutically appropriate dose levels (71). Experiments in dogs using the sedative drug and P-glycoprotein substrate acepromazine found that those dogs which were homozygous for the mutation showed increased sedative effects to IV acepromazine while dogs which were heterozygous for the mutation did not differ from genetically normal dogs (72).

As P-glycoprotein is normally expressed in liver and kidney, a lack of functioning p-glycoprotein can result in reduction of clearance of substrates. This is particularly relevant in canine cancer therapeutics, where there is a narrow therapeutic index, and reported case studies include dogs with ABCB-1 mutations undergoing chemotherapy for lymphoma which experienced more severe adverse effects than expected to P-glycoprotein substrates vincristine and doxorubicin (73, 74). A subsequent prospective study found that dogs that are heterozygous or homozygous for ABCB-1 mutation are more likely to experience neutropenia and thrombocytopenia than normal/wild type dogs (75).

It is proposed that the ABCB-1 mutation found in herding breeds arose in the early 1800s in Great Britain, in a common “founding” ancestor before the creation of breed registries (76). A recent evaluation of the prevalence of the mutation in the UK found that allelic frequency was highest for Rough (71 per cent) and Smooth (73 per cent) Collies, followed by Australian Shepherd dogs (46 per cent), Shetland sheepdogs (36 per cent), old English sheepdogs (11 per cent), and Border Collies (2 per cent), although the allele was not found in Bearded Collies. The study had concentrated primarily on herding breeds, and as only low numbers of other breeds had been used the presence of the allele in further breeds cannot be ruled out (77).

One relatively small US study of 100 banked feline samples found 5 animals with a two base pair deletion in the ADCB-1 gene, with one animal homozygous for this variant allele. This mutation results in a frameshift generating a series of stop codons immediately downstream from the deletion, resulting in a non-functional P-glycoprotein (78). However, breed data was not provided, and also as the samples derived from a limited population of cats in the catchment area of Washington State University Veterinary hospital; it is possible that this mutation is not yet widespread in the international feline population.

### Genes Involved in Pharmacodynamic Variation

Pharmacodynamics reflect a drug's ability to influence drug targets, normally a drug receptor or enzyme, and may involve both intended and unintended effects (2). Pharmacodynamic variation examples include genetic variation in expression levels in the target, such as with the human molecular target for warfarin, VKORC1 mentioned previously (58).

One example of a receptor polymorphism which has a strong effect on canine behavior is the gene encoding the oxytocin receptor OXTR. Oxytocin is considered to play an important role in human-animal interactions (79). A Swedish study, using Golden Retriever dogs and Eurasian wolves, identified polymorphisms located in the 3′ untranslated region immediately after the last exon of the dog OXTR-gene in both groups. One homozygous OXTR genotype (“AA”) was associated in dogs with increased human-directed social behavior in response to intranasal oxytocin, while the opposite was true of a second homozygous genotype (“GG”) (49). It is not clear as yet how this difference translates to therapeutic clinical practice.

One further polymorphism that may potentially be of importance in canine therapy relates to canine phosphodiesterase 5A (PDE-5A). The PDE-5 inhibitor sildenafil (Viagra) induces vasodilation through by increasing pulmonary vascular concentrations of cyclic guanosine monophosphate (cGMP), and is one of the recommended treatments for pulmonary hypertension (PH) in dogs (80). However, an exonic polymorphism has been identified in a range of canine breeds that alters the amino acid sequence of PDE-5 and is associated with significantly lower cGMP concentrations in healthy dogs that are homozygous for the polymorphism. This may explain the variability in response to sildendafil in dogs with PH (50).

### Genes Involved in “Pathway” Variation

In human medicine, statins are used primarily to reduce blood cholesterol and thereby reduce the risk of arteriosclerosis and adverse cardiac events. A lack of response may be attributed to a “pathway” pharmacogenetic difference. Statins target 3-hydroxy-3-methylglutaryl-coenzyme A (HMG-CoA) reductase, the enzyme involved in the biosynthetic pathway for cholesterol, leading to a decreased concentration of cholesterol in the cell. This lowering of intracellular cholesterol normally stimulates the synthesis of the LDL receptors (81) but with homozygous familial hypercholesterolemia, a condition caused by mutations in the low-density lipoprotein receptor (LDLR), patients may respond poorly or not at all to statin therapy (82).

As of yet, the authors can find no clear example of a specific “pathway” pharmacogenetic difference in veterinary species.

### Genes Involved in “Off-Target” Variation

The exemplar for an “off-target” effect in human medicine is the case of the major histocompatibility class I human leukocyte antigen-B (HLA-B) and the antiviral drug abacavir. Candidates for treatment with abacavir must be tested for the allele HLA-B^*^5701, as this allele is associated with idiosyncratic drug reactions to abacavir which presents in the first 6 weeks of treatment, includes skin rash and fever, and can be severe and life-threatening (83). Abacavir binds the peptide-binding groove of HLA-B^*^57:01, allowing the presentation of peptides that normally cannot be bound by the HLA-B complex (84).

In veterinary medicine, there are a number of well-known idiosyncratic responses to drugs, many of which present as immune-mediated reactions (85). Both feline (86) and canine (87) histocompatibility complex genes are known to be polymorphic: it is likely that future research will identify the alleles associated with immune-mediated drug reactions.

Malignant hyperthermia (HM) is a rare inherited hypermetabolic condition caused by an uncontrolled elevation of skeletal muscle cytoplasmic calcium ions, leading to strong muscle contractures, metabolic and respiratory acidosis, and dangerously elevated body temperature (31). As described previously, the study of MH was accelerated by recognition of the porcine presentation of MH, which allowed the development of the pig experimental animal model. It is now understood that depolarising muscle relaxants such as succinylcholine trigger MH, and also inhalant anesthetics other than nitrous oxide (88). Volatile anesthetics induce their effects through acting on a range of targets in excitable tissue, although the intended targets are voltage-gated channels, excitatory receptors and inhibitory receptors in the brain and spinal cord. Although the mechanism is not fully understood, all manifestations of MH are considered associated with some form of autosomal dominant variation in the ryanodine receptor RYR-1. MH has been documented in humans, pigs, horses, dogs, and cats (89). Porcine MH presents clinically in a very similar fashion to human MH but develops much more rapidly. The mutation identified in pigs consists of a cysteine for arginine transposition in the RYR-1 gene (30). MH has also been identified in horses, and presents very similarly to MH in humans, evolving over 3–4 h of anesthesia (89). The mutation found in horses to date is a missense base substitution in the RYR-1 gene which results in an amino acid substitution (54). In dogs, the RYR-1 mutation has been identified as a T to C substitution that changes an amino acid codon from valine to alanine (V547A) in a highly conserved region of the canine RYR-1 gene (53). Although specific breeds have been proposed to present more frequently with MH, all dogs are considered potentially susceptible (89).

## Translating PGx to the Clinic

**Figure 1:**
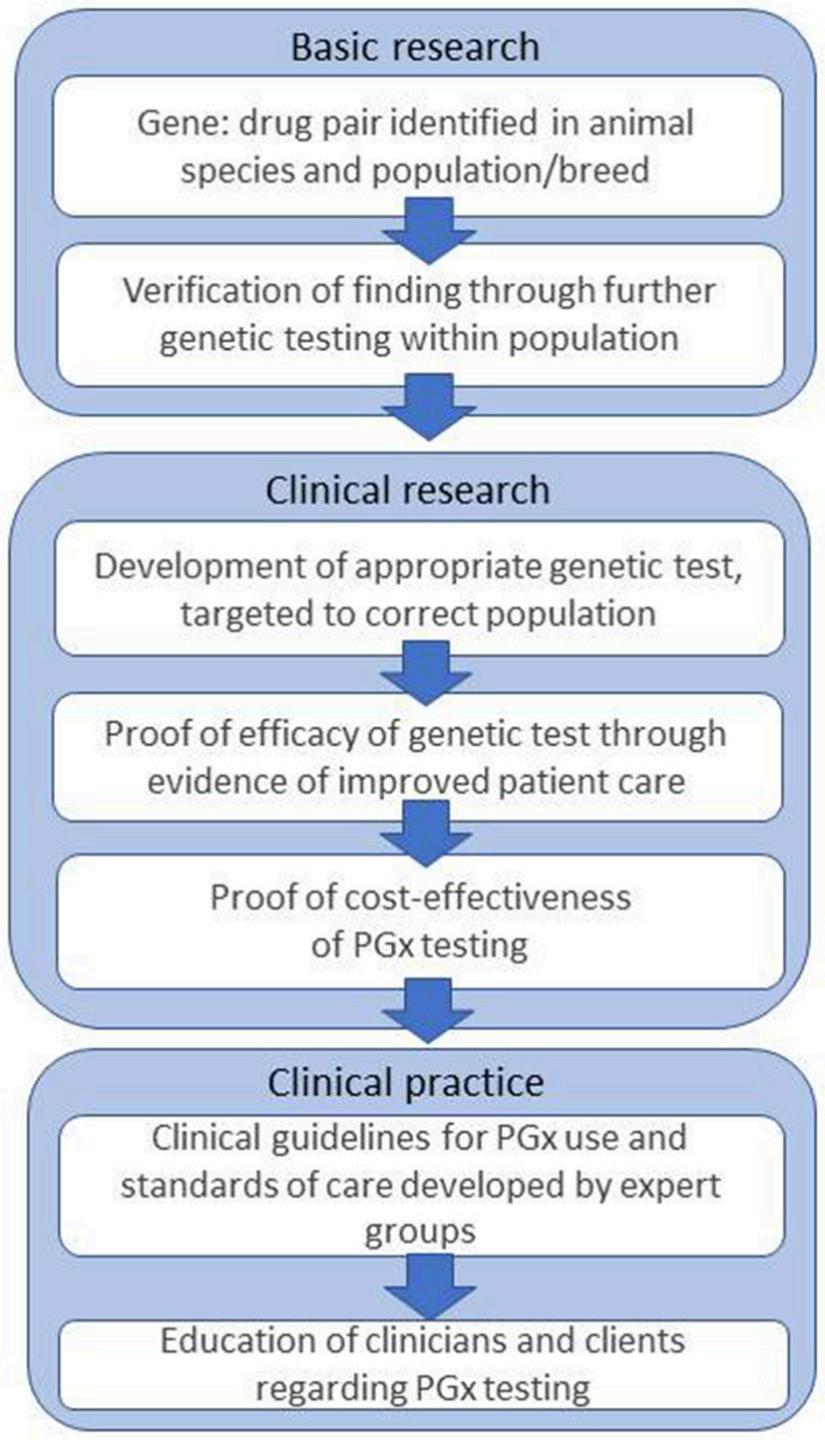
Steps and challenges in bringing PGx to the veterinary clinic, based on Figure 1 from Swen et al. (3).

In human medicine, the challenges and obstacles which act as barriers to implementation of PGx have been discussed for more than 10 years, and are still ongoing (3, 90–92). The steps are outlined in Figure 1.

The outputs from basic and clinical research have resulted in the identification of a significant number of “clinically actionable” pharmacogenes. One methodology which has added significantly to PGx knowledge is genome-wide association studies (GWAS) (93). This technique used microarrays of millions of SNPs, large genome databases, and statistical computing to determine the probability that specific variants are associated with a disease, or with an ADR. PGx examples found by GWAS include variants in *CYP2CI9* and resulting guidance of the dose of clopidogrel, and also variants in interleukin 2B and guidance on use of interferon-α (94).

In the European Union, medicines may be authorized by either an individual member state or via a centralized authorization procedure administered by the European Medicines Agency. More than 15% of the 517 human medicines that the European Medicines Agency has evaluated since central authorization commenced in 1995 include PGx information in the advisory documentation (95). In the USA, where there is a single authorization authority for medicines, the Food and Drug Administration (FDA) has added PGx information to over 200 authorized drug products used in human medicine, in some cases including recommendations for specific genetic tests to pre-screen patients before specific drugs are used (70). As an example, the FDA's “boxed” warning on the package insert of the anticonvulsant drug carbamazepine, it recommends that prior to prescribing physicians should screen patients of Asian ancestry for the MHC-I human leukocyte antigen-B HLA-B^*^1502 allele, due to the increased risk of fatal dermatologic reactions, including toxic epidermal necrolysis (TEN) and Stevens-Johnson syndrome (SJS) (96).

Despite this information, from USA survey results published in 2012 (97) and a further European survey in 2017 (98), human physicians have an awareness of PGx, but on the whole, they do not yet feel confident in use of PGx data. Moreover, the addition of such data by regulatory bodies has not been without consequences in human medicine. When screening for HLA-B^*^1502 was introduced as a routine service by the Hong Kong medical services, new prescriptions for carbamazepine declined dramatically overall despite only 17% of the tested population proving to be HLAB^*^15:02 positive, as clinicians chose drugs that did not require genetic testing. Instead, prescriptions for other drugs increased, one of which was phenytoin, a drug structurally similar to carbamazepine and which is also associated with TEN/SJS but which at the time did not have an FDA warning relating to HLA-B^*^1502 (99).

In human medicine, further challenges to the clinical use of PGx data include lack of evidence for improved patient care through PGx testing and perceived lack of guidelines for translating genetic test results into clinical decision-making (3). To begin to address this, a significant tool for dissemination of PGx information to physicians has been the creation of the Pharmacogenomics Knowledgebase (100). This is an online, freely available resource that collects and disseminates evidence-based human PGx information and currently lists and provides detailed information, including links to supporting literature on 65 “Very Important Pharmacogenes” (VIPs) in humans, the latter having been chosen following an extensive review of FDA pharmacogenetic information from and Clinical Pharmacogenetic Implementation Consortium (CPIC) nominations (100).

The sheer volume of data now presented to the clinician means that electronic medical recording systems (EMR) combined with computerized clinical guidance must form a key element in the realization of personalized medicine (101) and therefore the most exciting development in human PGx is the developing linkages between patient EMR and PGx data. In the US, EMR systems have been in widespread use for over a decade (102). In Europe, where EMR systems are in place at a national level in a number of EU states, combined with social welfare-based healthcare, there is considerable potential to link patient information with prescribing practice. In 2015, the Council of the European Union adopted conclusions on personalized medicine, and invited member states to support efforts to bring personalized medicine to fruition (103).

The Ubiquitous-Pharmacogenomics (U-PGx) Consortium is an example of a recent ongoing EU-funded collaborative effort to bring PGx to clinical decision-making across Europe. This project arose from work initially carried out by the Dutch Pharmacogenomics Working Group (DPWG) (5). The realization of the U-PGx project relies on a complex IT support system for integration of PGx test results with EMR systems and clinical decision support systems. Such EMR systems are currently operational in several, but not all EU countries, and for this project, seven implementation countries will be Austria, Great Britain, Greece, Italy, Netherland, and Spain. Once IT resources, guidelines, and training are in place, the second phase involves pre-emptive genotyping of >8,000 patients using a panel of 50 variants in 13 pharmacogenes into clinical practice, as part of a large prospective, international, block-randomized, controlled study named PREPARE (PREemptive Pharmacogenomic testing for preventing Adverse drug REactions) (5). The third phase will focus on expanding on and integrating knowledge of the influence of genetic variation on drug response, while the fourth phase will focus on ethical and legal issues associated with PGx implementation (104).

The later processes will also include a cost-benefit analysis of the utility of large-scale pharmacogenomic testing. Cost-effectiveness of PGx is an ongoing concern in human medicine (3, 91), although a recent meta-analysis has concluded that in a future where personal genomic information is available as part of normal EMR, the majority of economic evaluations would find PGx to be cost-effective (105). In countries where healthcare is primarily funded through taxes, the final decision-making on the utility of PGx testing will be taken by governmental health departments, and therefore human medical economics do not translate directly to veterinary medical economics. In veterinary medicine, costs associated with DNA testing are borne primarily by the animal owner, and data are likely to be held within privately-owned corporate databases. In comparison with human medicine, progress in PGx-driven prescribing in veterinary medicine has been slow to date, although, from a recent, critical and bioethically focused opinion piece published in Nature, pet genetic testing has become a “booming industry” and at least one US corporate veterinary hospital chain recommends genetic testing in order to “enable proactive, individualized healthcare” (106).

Current progress in the steps toward the clinic in veterinary medicine appears to be at an early “clinical research” stage, although international collaborative efforts are becoming visible in the domain of animal genetic testing (59). Currently, there are several information sources regarding genetic diseases in veterinary species, which include PGx variation. The most extensive is the website Online Mendelian Inheritance in Animals (OMIA), an academia-led and evidence-based site (107). This site provides a catalog of inherited traits in a broad range of animal species and includes pharmacogenes, however most of the pharmacogene findings reported are based on small-group studies. Genome-wide association studies are being used to identify genetic loci associated with disease in veterinary species, but as yet these have been limited in comparison with human studies, and also these have not focussed on PGx differences (106). This means very little statistically robust PGx data is currently available to veterinary clinicians, and commercially available PGx tests appear to be currently limited to two tests in dogs: one form of the ABCB-1 (listed as MDR-1) mutation, and one form of malignant hyperthermia (RYR-1) mutation. At the current state of veterinary genomics databasing, any claim by veterinary businesses that they can currently provide personalized medicine does not stand up to close scrutiny, nor can the majority of tests available in veterinary medicine truly guide therapy for improved health. The one group of animals for which pharmacogenetic testing should be considered “standard of care” are those canine breeds with a high risk of ABCB-1 mutation, particularly when treatment with vincristine, vinblastine, doxorubicin, loperamide, or a macrocyclic lactone is proposed (108, 109).

Pet genetic testing is unregulated, unlike human genetic testing in many jurisdictions. For quality assurance, the World Health Organization advocates regulatory oversight of genetic testing, and provides a benchmark regarding what such quality assurance should look like, from the point where the clinician decides whether a test is warranted, to the validity of the test, the accreditation of the laboratory and the interpretation of the results combined with counseling of the client/patient (110). Although this is a broader discussion than PGx, as pet genetic databases expand, there will need to be ongoing discourse regarding the ethics in and around the data generated: where and how long the data is to be stored, who is to be responsible for data sharing, how will data on individual animals affect insurance and the responsibility and/or potential liability of dog breeders toward owners (106).

Despite all of these concerns, there remains genuine scope for advances in PGx as genetic databases expand, with real scope for collaborative efforts between the private genetic companies and independent academic veterinary researchers to solve therapeutic problems and differences in responses. Owner investment in genetic tests, with permission, may serve as a form of “crowd-funding” for genome-wide association studies, and in addition, the potential for the databases to provide insight into disease processes may permit funding under the “one-health” banner.

## Conclusion

Human medicine stands at the brink of realization of personalized medicine, with plans in place at European Health policy level to deliver real outcomes by 2025. As personalized human medicine evolves, the pet owners will expect veterinary medicine to follow suit, but the risk is that without regulation, validation and international collaboration, personalized pet PGx data will be limited and primarily within the ownership of private genetic industry. Despite this risk, there are real opportunities to improve animal health, if owners, clinicians, genetic companies, and researchers work together with the goal that both animals and humans may benefit from this genomic revolution.

## Author Contributions

DC designed and wrote the paper. FD supplied the idea, critically reviewed the content, and proposed changes. Both authors approve publication of the content.

### Conflict of Interest Statement

The authors declare that the research was conducted in the absence of any commercial or financial relationships that could be construed as a potential conflict of interest.
